# The Pentose Phosphate Pathway Dynamics in Cancer and Its Dependency on Intracellular pH

**DOI:** 10.3390/metabo10070285

**Published:** 2020-07-11

**Authors:** Khalid O. Alfarouk, Samrein B. M. Ahmed, Robert L. Elliott, Amanda Benoit, Saad S. Alqahtani, Muntaser E. Ibrahim, Adil H. H. Bashir, Sari T. S. Alhoufie, Gamal O. Elhassan, Christian C. Wales, Laurent H. Schwartz, Heyam S. Ali, Ahmed Ahmed, Patrick F. Forde, Jesus Devesa, Rosa A. Cardone, Stefano Fais, Salvador Harguindey, Stephan J. Reshkin

**Affiliations:** 1 Alfarouk Biomedical Research LLC, Temple Terrace, FL 33617, USA; 2 American Biosciences Inc., New York, NY 10913, USA; dwales@americanbiosciences.com; 3 Al-Ghad International College for Applied Medical Sciences, Al-Madinah Al-Munawarah 42316, Saudi Arabia; 4 College of Medicine, University of Sharjah, Sharjah P. O. Box 27272, UAE; samahmed@sharjah.ac.ae; 5 The Elliott-Elliott-Baucom Breast Cancer Research and Treatment Center, Baton Rouge, LA 70806, USA; drrobertelliott@cox.net; 6 The Sallie A. Burdine Breast Foundation, Baton Rouge, LA 70806, USA; amanda25304@live.com; 7 Clinical Pharmacy Department, College of Pharmacy, Jazan University, Jazan 45142, Saudi Arabia; Ssalqahtani@jazanu.edu.sa; 8 Institute of Endemic Diseases, University of Khartoum, Khartoum 11111, Sudan; Mibrahim@iend.org (M.E.I.); Derma55@yahoo.com (A.H.H.B.); 9 Department of Clinical Laboratory Sciences, Faculty of Applied Medical Sciences, Taibah University, Al-Madinah Al-Munwarah 42353, Saudi Arabia; Shoufie@taibahu.edu.sa; 10 Unaizah College of Pharmacy, Qassim University, Unaizah 56264, Saudi Arabia; gamaosma963@gmail.com; 11 Assistance Publique des Hôpitaux de Paris, 75003 Paris, France; dr.Laurentschwartz@gmail.com; 12 Department of Pharmaceutics, Faculty of Pharmacy, University of Khartoum, Khartoum 11111, Sudan; Heyam57@hotmail.com; 13 Department of Oesphogastric and General Surgery, University Hospitals of Leicester, Leicester LE5 4PW, UK; Ahmed.ahmed@doctors.org.uk; 14 CancerResearch@UCC, Western Gateway Building, University College Cork, Cork T12 XF62, Ireland; p.forde@ucc.ie; 15 Scientific Direction, Foltra Medical Centre, Travesía de Montouto 24, 15886 Teo, Spain; jesus.devesa@usc.es; 16 Department of Biosciences, Biotechnologies, and Biopharmaceutics, University of Bari, 90126 Bari, Italy; rosaangela.cardone@uniba.it (R.A.C.); stephanjoel.reshkin@uniba.it (S.J.R.); 17 Department of Oncology and Molecular Medicine, Istituto Superiore di Sanità, Viale Regina Elena 299, 00161 Rome, Italy; stefano.fais@iss.it; 18 Department of Oncology, Institute for Clinical Biology and Metabolism, 01004 Vitoria, Spain; salvaszh@telefonica.net

**Keywords:** cancer, metabolism, enzyme, pH, redox

## Abstract

The Pentose Phosphate Pathway (PPP) is one of the key metabolic pathways occurring in living cells to produce energy and maintain cellular homeostasis. Cancer cells have higher cytoplasmic utilization of glucose (glycolysis), even in the presence of oxygen; this is known as the “Warburg Effect”. However, cytoplasmic glucose utilization can also occur in cancer through the PPP. This pathway contributes to cancer cells by operating in many different ways: (i) as a defense mechanism via the reduced form of nicotinamide adenine dinucleotide phosphate (NADPH) to prevent apoptosis, (ii) as a provision for the maintenance of energy by intermediate glycolysis, (iii) by increasing genomic material to the cellular pool of nucleic acid bases, (iv) by promoting survival through increasing glycolysis, and so increasing acid production, and (v) by inducing cellular proliferation by the synthesis of nucleic acid, fatty acid, and amino acid. Each step of the PPP can be upregulated in some types of cancer but not in others. An interesting aspect of this metabolic pathway is the shared regulation of the glycolytic and PPP pathways by intracellular pH (pHi). Indeed, as with glycolysis, the optimum activity of the enzymes driving the PPP occurs at an alkaline pHi, which is compatible with the cytoplasmic pH of cancer cells. Here, we outline each step of the PPP and discuss its possible correlation with cancer.

## 1. Introduction

In 1935 and 1936, Otto Warburg showed that pyridine nucleotide diphosphopyridine nucleotide DPN (currently known as NAD^+^) functioned as an electron carrier [1,2,3]. He also demonstrated the presence of a second enzyme called triphosphopyridine (TPN), currently known as NAP^+^. He then discovered the Zwischenferment enzyme (known as glucose-6-phosphate dehydrogenase (G6PDH)) [1,2]. Many decades later, the Pentose Phosphate Pathway (PPP) was introduced to biochemistry.

The PPP has also been termed the phosphogluconate pathway or the hexose monophosphate shunt. It is a cytoplasmic pathway consisting of two phases: (i) the oxidative phase, followed by (ii) the nonoxidative phase. This pathway finishes as one of the glycolytic intermediates, e.g., Glyceraldehyde 3-phosphate. The oxidative phase produces two molecules of NADPH, while the nonoxidative phase produces a building block of nucleic acid.

**Figure 1:**
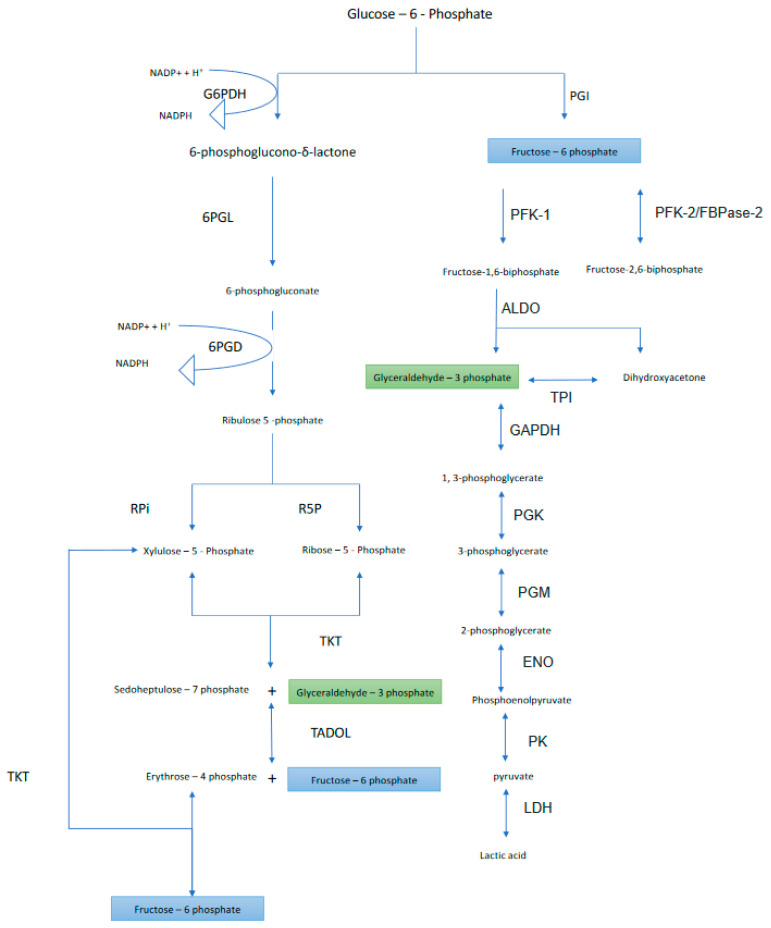
Scheme showing the interaction between glycolysis and PPP.

When glucose enters the cell, glucokinase phosphorylates the free glucose in order to anchor it inside the cell by forming glucose-6-phosphate. Glucose-6-phosphate has two fates: either (i) to convert to fructose-6-phosphate via glucose-6-phosphate isomerase and complete the classical glycolytic pathway, or (ii) to convert to 6-phosphoglucolactone via glucose-6-phosphate dehydrogenase (G6DPH) and complete the Pentose Phosphate Pathway. We previously discussed how cancer cells recruit or manipulate the glycolytic pathway [4]. In the current manuscript, we will discuss the latter fate of glucose-6-phosphate conversion and its effects on cancer (see Figure 1).

## 2. Oxidative Phase

### 2.1. First Step

Upon glucose fixation, glucose-6-phosphate is converted to 6-phosphoglucolactone via glucose-6-phosphate dehydrogenase (G6DPH), which requires NADP^+^ to become NADPH (Figure 1). This step is irreversible and is catalyzed by G6PDH, which has two subtypes: A and B. While there is not a strong requirement for Mg^+2^ (it has not been demonstrated to be an absolute requirement for the Leuconostoc enzyme), it is slightly stimulated by HCO^−3^ [5,6]. Indeed, the optimum activity of G6PDH occurs at an intracellular alkaline pH (pHi) which is compatible with the cytoplasmic pH of cancer cells [7,8].

The activity of G6PDH is enhanced by spironolactone [9], and insulin [10]; however, whether dexamethasone and glucose alone can increase G6PDH activity or not remains controversial [10,11]. It has been shown that high levels of glucose inhibit G6PDH activity [12] and reduce NADPH. However, such data is controversial, since Sodium Hydrogen Exchanger (NHE) stimulates glycolysis and, therefore, may act as a transient inhibitor since NADPH can also increase the Pentose Phosphate Pathway (PPP).

A study by Catanzaro et al. demonstrated G6PDH overexpression in cisplatin-resistant cancer cells, and that the use of G6PDH competitive inhibitor, 6-aminonicotinamide-AN6, sensitized the cells to cisplatin [13]. The blockade of G6PDH induced the lapatinib (TK inhibitor)-mediated cytotoxicity via enhancing autophagy [14]. Moreover, obstructing G6PDH function reversed cisplatin resistance via interfering with the homeostatic balance of reactive oxygen species (ROS), resulting in less available NADPH to neutralize ROS [15].

Possible inhibitors of G6PDH include P53 [16], ATP, which inhibits G6PDH both directly [17] and by increasing the NADPH/NADP^+^ ratio [18], and Palmitoyl-CoA which also inhibits G6PDH [19]. Moreover, NAD^+^ increases G6PDH activity [20]. Several nutraceutical products have been shown to inhibit G6PDH and could be used therapeutically, like Fermented wheat germ extract (FWGE) (Metatrol^®^, Avemar^®^, Polydatin or Piceid (major Resveratrol derivative) [21,22,23].

#### Evolutionary Consequences of Population Selected G6PDH Deficiency

Interestingly, G6PDH deficiency is an X-linked recessive hereditary disease [24,25]; this deficiency is accompanied by several clinical manifestations, as follows:

G6PDH deficiency confers natural resistance against malaria [26].
  From an epidemiological standpoint, G6PDH deficiency is correlated with reduced cancer risk [27,28,29]. Thus, G6PDH deficiency represents a natural prevention strategy against tumor development. Such a deficiency could be explained as follows:
Free radical scavengers such as NADPH are cytoplasmic reducing agents (see below). In other words, NADPH is a cytoplasmic free radical scavenger. Overproduction of free radicals, reactive oxygen species (ROS) and reactive nitrogen species (RNS) by NAD(P)H oxidase isoforms and NO synthase respectively, induces cellular senescence and apoptosis [30], as well as necrosis [31].
Cancer cells induce anti-apoptotic proteins [32] and are devoid of pro-apoptotic protein expression [33]. Hence, the ratio of the anti-apoptotic/apoptotic proteins is indicative of increased malignancy [34]. NADPH increases this ratio and, therefore, decreases apoptosis [35,36]. Also, the pro-apoptotic protein, BCL-2, has anti-oxidant properties [37], which increase the perturbations of the cellular redox status. So, two questions arise: is it preventive or prompting? And does it depend on the stage of the tumor?

### 2.2. Second Step

As shown in Figure 1, 6-phosphoglucono-δ-lactone is converted into 6-phosphogluconate via the 6-phosphogluconolactonase (6PGL) enzyme.

The activity of 6-phosphogluconolactonase enzyme increases sharply as intracellular pHi increases [38], which is compatible with the cytoplasmic pH of cancer cells.

Epstein Barr virus (EBV) upregulates the expression of 6-phosphogluconolactonase [34], which is crucial to tumor growth [39]. Furthermore, it has been reported that 6PGL correlates with pancreatic cancer resistance to Gemcitabine [40].

The inhibitory abilities of an interesting compound, GP269, were tested against 6-phospho-gluconolactonase (6PGL). While this inhibition was attempting to target *Trypanosoma Brucei*, this compound could also be a potential therapeutic tool for breast cancer, since 6PGL was found to be overexpressed in human epidermal growth factor receptor 2 (HER2) positive breast cancer cells [41,42].

### 2.3. Third Step

In the last step of the oxidative phase, 6-phosphogluconate undergoes decarboxylation by the 6-phosphogluconate dehydrogenase (6PGD) enzyme in the presence of NADP^+^ to produce ribulose 5-phosphate and NADPH (Figure 1). NADPH inhibits 6PGD, so it has a negative mechanism. Again, the optimum pHi for this enzyme is alkaline [43,44].

Consistent with its association with poor prognoses, 6PGD supports tumor growth [39,45], metastasis [46], and chemotherapy resistance [47]. Aberrant expression levels of 6PGD were reported in cervical cancer and were linked to more proliferative and migratory abilities of cancer cells [48]. The deactivation of 6PGD resulted in a reduction of RhoA and Rac1 function, which, in turn, negatively affected the cancerous behavior of the cervical cancer cells [48]. Like G6PDH, 6PGD was reported to help in sensitizing cisplatin-resistant cells in both ovarian and lung cancer. In addition, 6PGD expression was shown to be linked with poor prognoses in breast cancer patients; thus, its inhibition was suggested to improve breast cancer outcomes [49]. Therefore, 6PGD overexpression is a successful adaptive (evolutionary) strategy to confer cancer cell survival. Therefore, it will not be surprising if there is extensive research on 6PGD as a potential target in cancer management [49]. Parietin (Physcione) is an anti-infectious agent [50] and a 6PGD inhibitor [45,51,52]. Therefore, it has been suggested that it be administered directly as an anticancer agent, as well as to resensitize resistant tumors to therapy [51,52,53]. This applies to solid tumors, but also to hematological malignancies, as it has been demonstrated that inhibition of 6PG sensitizes leukemia cells to the antimalarial drug, dihydroartemisinin [52]

## 3. Nonoxidative Phase

In contrast to the oxidative phase, in this phase, all the reactions are reversible. In accordance with the steps above, the nonoxidative steps are as follows.

### 3.1. Fourth Step

#### 3.1.1. Ribose-5 Phosphate Formation

Ribulose-5-phosphate produces Ribose 5-phosphate (R5P) via Ribose-5-phosphate isomerase (Rpi) (Figure 1). The formation of R5P depends on (i) cell growth [54], (ii) the redox state of the cell [55,56], and (iii) the metabolic state of the cell [3]. Moreover, Rpi has its optimum activity at an alkaline pHi [57].

Rpi has two forms: RpiA or RpiB [58,59], with RpiA being mainly expressed in humans [60] and RpiB mainly detected on lower microorganisms [61,62,63]. It has been shown that ribose-5-phosphate isomerase A overexpression promotes and is associated with several types of cancer, including liver, lung, and breast [64,65]. One of the possible explanations of how Rpi promotes carcinogenesis is through its activation of ERK and β-catenin pathways [64], and perhaps through the inhibition of LC3 (e.g., alteration of autophagy) [66].

#### 3.1.2. Xylulose 5-Phosphate Formation

Xylulose 5-phosphate (Xu5P) is formed from Ribulose-5-phosphate via the metalloproteinase enzyme Ribulose 5-Phosphate 3-Epimerase (RPE) (Figure 1), which has optimal activity at a slightly alkaline pHi [58].

Ribulose 5-Phosphate 3-Epimerase (RPE) has been detected in pancreatic cancer, and its effect might be through the activation of fructose 2,6 bisphosphate (F-2, 6BP), which activates phosphofructose kinase 1 (PFK1), and/or K-ras [67,68,69].

Hydrogen peroxide (H_2_O_2_) inactivates RPE, while iron and manganese activate the RPE [70]. It is known that a high glucose load induces intracellular alkalinity and the Warburg Effect [71]. Also, in response to higher glucose, Xu5P activates the carbohydrate-responsive, element-binding protein (ChREBP) [72,73], which is also known as MLX-interacting protein-like (MLXIPL) and, in humans, is encoded by the MLXIPL gene [74]. ChREBP protein is overexpressed in cancer, and its attenuation is accompanied by the activation of P53 and induction of cell cycle arrest [75,76,77]. Moreover, ChREBP mediates the activation of L-Pyruvate Kinase (L-PK), which is essential for pyruvate formation [78,79].

### 3.2. Fifth Step

Here, ribose-5-phosphate and xylulose-5-phosphate interact to form sedoheptulose 7-phosphate to produce sedoheptulose 7-phosphate and glyceraldehyde 3-phosphate using transketolase enzyme (TKT) (Figure 1). Glyceraldehyde 3-phosphate is an essential intermediate to induce glycolysis and the Warburg Effect [4].

Transketolase (TKT) is a thiamine-dependent enzyme [80], again having optimal activity at an alkaline pHi [81,82]. TKT plays a crucial role in regulating the cellular redox state [83]. TKT expression is associated with poor prognoses and metastases, while its suppression decreases tumor metastasis [84]. Oxythiamine is an inhibitor of TKT [85]. Moreover, it has been shown that Furosemide (Lasix^®^) have a drastic inhibitory effect on TKT, as it induces thiamine deficiency [86].

### 3.3. Sixth Step

Sedoheptulose 7-phosphate interacts with glyceraldehyde 3-phosphate to produce erythrose 4-phosphate and fructose 6-phosphate via the transaldolase enzyme (TADOL) (Figure 1), which has optimal activity at alkaline pHi [87,88].

The TADOL enzyme is a rate-limiting enzyme in the nonoxidative PPP [89,90,91]. *Transaldolase 1 gene (TADOL1)* is ubiquitously expressed, except in erythrocytes (Red Blood Cells (RBCs)), that might be challenging, because RBCs rely on the PPP in their metabolism.

It had been shown that the TADOL activity was higher in liver tumor rather than in normal control rat liver [90]. TADOL is a master regulator of the redox state of the cell, and so its overexpression might affect not only tumor survival, but also response to therapy. It has been shown that elevated TADOL expression correlates with a decrease in response to HER2 inhibition in breast cancer patients [92].

Starvation decreased TADOL activity, which is beneficial to the prevention and/or slowing of cancer development [90,93,94]. Arabinose 5-phosphate is the aldopentose (a metabolic intermediate in the biosynthesis of lipopolysaccharide) inhibits TADOL [95].

The formation of fructose 1,6-bisphosphate inhibits TADOL activity, which might explain why the PPP and glycolysis do not occur simultaneously.

Some tissues, such as heart and skeletal muscle, have lower transaldolase protein expression [90]; as such, those tissues tend to have lower susceptibility for tumor growth. While we cannot conclude that the TADOL could be cancer’s Achilles’ Heel, it might open the window regarding PPP activity and tissue susceptibility; this might, in turn, reveal why PPP favors carcinogenesis. Also, these tissues have a higher mitochondrial number compared to other tissues, and so, we propose that PPP activity might be inversely proportional to mitochondrial metabolism.

### 3.4. Seventh Step

This step could be represented as reinventing the wheel to closely pool glycolytic pathways, like the fifth and sixth steps. In this step, xylulose 5-phosphate interacts with erythrose 4-phosphate to produce glyceraldehyde 3-phosphate and fructose 6-phosphate via transketolase (TKT), which has optimal activity at pHi of 7.5–7.6 [90,96] (Figure 1). For TKT kinetics, see above.

It seems that the net result of the second phase of the PPP (i.e., the nonoxidative phase) is to support the glycolytic pathway via the provision of two molecules of fructose-6-phosphate and one of glyceraldehyde-3-phosphate. These molecules are critical intermediates in the glycolytic pathway. Therefore, in conclusion, the PPP provides the nucleic acid building blocks, maintains the redox state of the cell and supports cellular energy.

#### 3.4.1. NADPH and GSH

**Figure 2:**
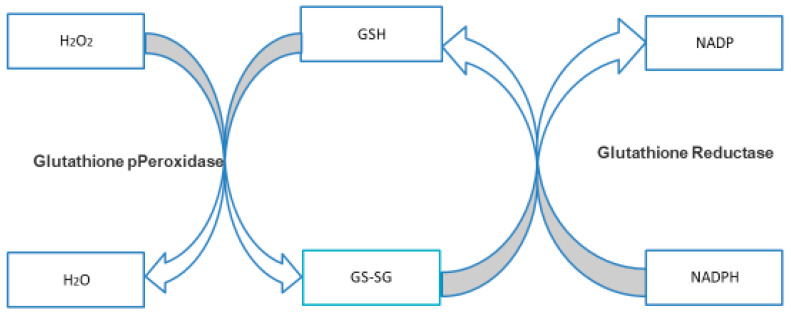
Glutathione Reductase Cycle.

How does the PPP maintain oxidation-reduction reactions in the cell (i.e., the Redox state of the cell)? During the first and the third steps (see above), nicotinamide adenine dinucleotide phosphate (NADP^+^) is a cofactor in the formation of NADPH; that is, each round of the PPP forms two molecules of NADPH. NADPH acts as a hydrogen donor to the disulfide bond of the oxidized glutathione (G-S-S-G) bond to form reduced glutathione GSH, while the NADPH becomes NADP^+^ via the glutathione reductase enzyme. Then, GSH donates its H^+^ to the oxidant, e.g., H_2_O_2_, to produce H_2_O, while the GSH converts back into the oxidized form, G-S-S-G, via the activity of glutathione peroxidase enzyme. Therefore, this system acts like a series of gears in that the NADPH gear activates the glutathione gear and is a scavenger of free radicals “Glutathione Redox Cycle” (Figure 2) [97,98,99,100]. Therefore, the NADP^+^/NADPH ratio could be expressed indirectly as the GSH/GSSG ratio. If the PPP decreases, NADPH decreases, resulting in an abundant glutathione ratio in the oxidized form.

#### 3.4.2. The Overview of PPP and Cancer

Reactive oxygen species (ROS) and reactive nitrogen species (RNS) are double-edged swords. On the one hand, they lead to aberrant gene expression and genetic instability that are fundamental processes in the development and progression of cancer [101,102,103,104]. Furthermore, ROS and NOS, at a certain point, are supportive of evolutionary tumor trajectory via supporting angiogenesis and metastasis, especially during cellular senescence, as they are hallmarks of inflammatory and tumor microenvironments [105,106,107,108]. However, the presence of ROS and RNS is a key determinant in the apoptosis of cancer cells [109,110,111,112,113]. Therefore, cancer cells robustly adjust to a very fine ratio of the cellular redox state in order to confer their survival. As a result, we conclude that highly dividing cells are well equipped for tumor development, while senescent cells are more prone to induce metastasis, as these cells lack an efficient PPP [39,114]. This inefficiency during senescence is not accompanied by a decrease in the level of metabolites since metabolites can derive from two sources: (i) the metabolites of the nonoxidative phase, e.g., ribose 5-phosphate, ribulose, xylulose 5-phosphate and sedoheptulose 7-phosphate [115], and (ii) those coming from glycolysis rather than the oxidative phase of the PPP, which reflects the activity of the PPP on the next stage of metastasis and settlement at the distal site (Mesenchymal Epithelial transition (MET)) [116,117]. In other words, cancer cells manipulate and escalate the metabolic pathways, and the PPP is one of those pathways to support their evolutionary fitness.

The Pentose Phosphate Pathway maintains cancer’s redox state, but it also affects cell signaling during the cell cycle, as well as managing the metabolic pathways, e.g., xylulose 5-phosphate (see above) [72,117,118,119,120].

Necrosis is another cellular death pathway that has a drastic effect on tumorigenesis. That is why chemotherapy acts via apoptosis and not necrosis [121,122,123,124,125]. However, several mechanisms of drug resistance are associated with the upregulation of the PPP to prevent the formation of free radicals [126]. The role of the PPP is not confined only to antagonizing the effect of chemotherapeutic agents; it also increases the expression and/or activity of the proteins (e.g., Multidrug-resistance-associated protein (MRP), ATP-binding-cassette (ABC) transporters) that either promote the efflux of anticancer drugs (e.g., vincristine, doxorubicin, daunorubicin) or block the influx of these agents [127,128,129].

#### 3.4.3. The Possible Crosstalk between the Glycolysis and the Pentose Phosphate Pathway (NAPDH Is a DoublE-edged Sword)

Generally, the presence of NADPH inhibits G6PDH, thereby blocking the PPP negative feedback control) and might block the possibility of nucleic acid pooling. Again, this raises an important question: does NADPH suppress tumor growth? NADPH has a critical role in the completion of the first phase of glycolysis (preparatory phase). Therefore, NADPH shifts the metabolism from PPP to glycolysis. The formation of NADPH will increase the formation of NAD+, and therefore, support the formation of pyruvic acid and lactic acid, which represents an evolutionary advantage [130]. Therefore, the role of NADPH in cancer is a conundrum.

In aggressive cancer cells, where the metabolic need for nucleotides exceeds that of NADPH, TKT and TALDO catalyze the reverse reactions and divert glyceraldehyde 3-phosphate and fructose 6-phosphate from glycolysis to the nonoxidative PPP to produce additional ribonucleotides. In this regard, glycolysis itself acts as a backup for the DNA building blocks. Therefore, the nonoxidative phase might represent the bridge between phase 1 of the PPP and phase 1 of glycolysis, that could enhance the metabolic plasticity of cancer cells [131]. An important overlap between these two systems that has, to our knowledge, been overlooked is that both pathways share an exquisite sensitivity to cytoplasmic, intracellular pH (pHi), in that all the enzymes that drive both pathways have their optimum activities at alkaline values (Table 1). This probable parallel regulation by processes that determines pHi underlines the importance of both metabolic pathways in cancer and of the aforementioned crosstalk of phase 1 of the two systems in determining the relative usage of one or the other pathways in a specific cellular context. Indeed, this shared intracellular alkaline optimum of the enzymes that drives glycolysis and the PPP in cancer cells opens new avenues for possible therapeutic opportunities for manipulating this pathway via its sensitivity to changes in pHi. Additionally, the formation of fructose 1,6-bisphosphate inhibits TADOL activity, which might further explain why the PPP and glycolysis do not occur simultaneously.

## 4. Concluding Remarks

Cancer as a biological system and/or tissue has unusual physiological, biochemical, and biophysical parameters compared to normal tissues [134]. To maintain viability, cancer has modified several existing metabolic pathways to fulfill its energy demands with as low a cost as possible [135]. One of these metabolic pathways that utilize glucose in the cytoplasm is the Pentose Phosphate Pathway (PPP). In the 1920s, Otto Warburg observed that cancer cells had a higher fermentation rate, and later concluded that cancer relied on the cytoplasmic utilization of glucose rather than mitochondrial utilization. However, cytoplasmic utilization can occur through glycolysis and/or the PPP. In our previous work, we showed the detailed biochemistry of glycolysis in cancer and its possible therapeutic opportunities and limitations [4]. Here, we present a study of the PPP pathway that suggests that it has a higher impact on carcinogenesis than glycolysis, and that cancer might invest in the PPP rather than glycolysis based on the cellular state and the cell cycle phase, whether it undergoes proliferation or growth. Both glycolysis and the PPP have mutual intermediates, but the PPP has more ramifications through its contribution to the synthesis of nucleic acids and maintains the redox state at the optimum that assures cancer cell survival. Also, the PPP interacts with the mitochondrion and so manipulates the programmed cell death pathway (apoptosis). Therefore, the PPP is paramount in cancer biology. Much work is now focusing on targeted diagnostics and therapeutics by selecting definite steps; however, tumor colonies consist of a heterogeneous population having different modified metabolic strategies that can confound the diagnosis and therapy of cancer. We have tried here to contribute to drawing a complete picture of the cancer puzzle to be in a relatively better position for its management rather than its eradication. Lastly, the described intracellular alkaline optimum of all the enzymes that drive the PPP in cancer cells (Table 1) opens new avenues for understanding the roles, regulations, and possible therapeutic opportunities for manipulating this pathway via its sensitivity to the changes in pHi that is common to cancer cells.

## Figures and Tables

**Table 1:** Optimum pHi for each PPP driving enzyme.

Enzyme	Optimum pH
Glucose-6-phosphate dehydrogenase (G6PDH)	7.8 [8]
6-phosphogluconolactonase (6PGL)	7.4 [38]
6-phosphogluconate dehydrogenase (6PGD)	Range from (7–10) depending on several factors including the buffer used in the experiment [43]
Ribose-5-phosphate isomerase (RPi)	8.4 [132]
Ribulose 5-Phosphate 3-Epimerase (RPE)	7.25–7.5 [69]
Transketolase (TKT)	7.5–7.6 [82,90,96]
Transaldolase (TADOL)	8 [133]

## Abbreviations

G6PDH	Glucose-6-phosphate dehydrogenase
6PGL	6-phosphogluconolactonase
6PGD	6-phosphogluconate dehydrogenase
R5P	Ribose-5-phosphate isomerase
RPE	Ribulose 5-Phosphate 3-Epimerase
TKT	Transketolase
TADOL	Transaldolase
PGI	Phosphoglucose isomerase
PFK1	Phosphofructokinase-1
PFK2	Phosphofructokinase-2
ALDO	Fructose-bisphosphate aldolase
TPI	Triosephosphate isomerase
GAPDH	Glyceraldehyde phosphate dehydrogenase
PGK	Phosphoglycerate kinase
PGM	Phosphoglycerate mutase
ENO	Enolase
PK	Pyruvate Kinase
LDH	Lactate Dehydrogenase
